# Molecular Toxicity Virtual Screening Applying a Quantized Computational SNN-Based Framework

**DOI:** 10.3390/molecules28031342

**Published:** 2023-01-31

**Authors:** Mauro Nascimben, Lia Rimondini

**Affiliations:** 1 Department of Health Sciences, Center on Autoimmune and Allergic Diseases CAAD, Università del Piemonte Orientale, 28100 Novara, Italy; 2 Enginsoft SpA, 35129 Padua, Italy

**Keywords:** machine learning, in silico toxicity prediction, molecular fingerprints, spiking neural networks

## Abstract

Spiking neural networks are biologically inspired machine learning algorithms attracting researchers’ attention for their applicability to alternative energy-efficient hardware other than traditional computers. In the current work, spiking neural networks have been tested in a quantitative structure–activity analysis targeting the toxicity of molecules. Multiple public-domain databases of compounds have been evaluated with spiking neural networks, achieving accuracies compatible with high-quality frameworks presented in the previous literature. The numerical experiments also included an analysis of hyperparameters and tested the spiking neural networks on molecular fingerprints of different lengths. Proposing alternatives to traditional software and hardware for time- and resource-consuming tasks, such as those found in chemoinformatics, may open the door to new research and improvements in the field.

## 1. Introduction

Machine learning (i.e., ML) techniques provide data-driven tools to screen small molecules for quantitative structure–activity or property relationships (i.e., QSAR/QSPR), contributing to the preliminary selection of candidate compounds that will be validated experimentally by in vitro or in vivo assays [1]. ML QSAR models establish a connection between the structure of a molecule and its activity by regression if the property under investigation is continuous or by classification if the biological outcome is categorical [2]. Prioritizing the most suitable molecules for the goals of an experiment by virtual screening brings advantages in terms of time and resource-saving. Moreover, it can be carried out in a high-throughput fashion [3]. Machine learning algorithms previously tested on bioactivity data prediction included classic methodologies such as random forests [4,5,6,7], support vector machines [8,9,10], or k-nearest neighbors [11,12]; in addition, deep neural networks (i.e., NNs) were also employed due to their performance in examining complex libraries of compounds [13]. Spiking neural networks (i.e., SNNs) are different machine learning systems built around the concept of simulating biological processes by replacing the perceptron [14] with neuronal models borrowed from computational biology [15]. In recent years, the growing interest of the scientific community in these innovative networks led to the development of specific event-driven hardware to accomplish brain-inspired computations [16]. Pairing software based on spiking neural networks to neuromorphic computers, it could be possible to achieve low-power computations [17]. Together with quantum computing, neuromorphic platforms are alternatives to the standard clock-based hardware for bypassing the increasing demand of computational resources required by deep learning algorithms [18].

In ML QSAR, relating a molecule to a property requires encoding the molecule’s structural information into a learnable representation. After encoding, the molecular formula could be utilized in specialized software [19] or libraries [20] to extract descriptors acting as input features for ML models. These programs accept compounds portrayed as alphanumeric strings called simplified molecular-input line-entry systems (i.e., SMILES). However, recently several authors feed SMILES directly to their encoder–decoder models for property forecasting [21,22]: these models exploit the internal NN representations as latent features. Alternatively, the molecular structure could be represented in the form of graphs, non-Euclidean datatypes consisting of nodes and edges [23]; in that case, applying SMILES as starting data is beneficial because decoding the molecular graph directly may increase the complexity of the model [24]. Although implementing graph-based NN for property prediction is a popular choice still, a few problems affect this methodology: underfitting the train set [25], lack of performance on small datasets [26], over-smoothing [27], information loss while encoding the graph to a vector [28], and inability to preserve long-range node dependencies [29]. Due to these limitations, the classic ML model applied to molecular fingerprints still retains its validity, achieving remarkable results in QSAR studies [30,31,32]. Several of these ML protocols for virtual screening employ molecular fingerprints (i.e., MFs) as inputs rather than SMILES, substituting ASCII characters with sparse binary vectors collecting compounds’ features [33]. The MFs are sequences of bits illustrating the presence or lack of a specific molecular substructure; the length of the MFs is variable, ranging from 166 bits of the Molecular ACCess System (i.e., MAACS) [34] to 1024 of Daylight Chemical Information Systems [35] or 2048 bits using Morgan’s extended-connectivity fingerprints [36].

The current study aims to employ SNN for toxicity prediction using datasets of compounds converted into MAACS fingerprints. The choice of fingerprints as input data for the SNN is derived from the observation according to which binary vectors are the natural input of SNNs. Indeed, SNNs receive incoming information encoded as bit sequences that simulate neuronal spike trains [37]. The mechanism of the neurons constituting an SNN is similar to the activity in human neurons: the membrane potential is sensitive to over-threshold stimuli, causing modulations of trans-membrane ionic currents and the consequent passage of information to the following cell. The crucial aspect of SNNs compared to traditional NNs, is that not all information is transmitted to the next layer at every cycle, but only after certain conditions measured in terms of membrane voltages are met. In chemoinformatics, the application of SNNs is a novelty and this work may be the first attempt to promote SNN in QSAR toxicological investigations. The current work will test SNNs extensively in large databases of molecules with different characteristics; special attention will be paid to SNN parameter tuning, a practical aspect usually reported as challenging to handle [38]. This study will further relate chemoinformatics QSAR modeling to the novel field of neuroscience-inspired artificial intelligence [39].

## 2. Results

The results include the single dataset assessment by the SNNs (Section 2.1), SNN hyperparameter report for the best models (Section 2.2), and meta-analysis with the previous literature on the same data (Section 2.3). In Section 2.4, the SNN architectures studied for MAACS MFs were evaluated on more advanced MFs to judge the scalability of SNNs when providing inputs of different lengths.

### 2.1. Classification Outcomes

The results of the numerical experiments applying SNNs on each dataset for medicinal chemistry were reported in the following tables. Only the top five outcomes were included, with accuracy values reported as mean and standard deviation (i.e., Std) of the CV repetitions.

#### 2.1.1. Numerical Experiments on Clintox

The toxicity in clinical trials found by the U.S. Food and Drug Administration is the binary outcome (FDA-approved or not) of the Clintox benchmark. Classification outcomes of SNNs were collected in Table 1.

#### 2.1.2. Numerical Experiments on Tox21 NR-AR

The Tox21 dataset contains several measurements regarding toxicity by in vitro high-throughput screening. The NR-AR measures the anti-androgenic toxicity estimated in nipple retention. The SNN performance on this task is portrayed in Table 2.

#### 2.1.3. Numerical Experiments on Tox21 NR-ER-LBD

From Tox 21, the NR-ER-LBD variable determines the estrogen receptor nipple receptor binding site for transcriptional activity. It is an indicator of organ toxicity through the pathway for etonogestrel, a medication for birth control in women. The outcomes of the numerical experiments with SNN are delineated in Table 3.

#### 2.1.4. Numerical Experiments on Tox21 SR-ATAD5

In Tox21, the measurement SR-ATAD5 is not a toxicity target as a nuclear receptor but a quantity connected with the stress response pathway. It quantifies the genotoxicity captured by the ATAD5 signaling pathway as part of the stress response panel activated when a cell detects DNA damage. Classification outcomes from SNNs could be found in Table 4.

#### 2.1.5. Numerical Experiments on TOXCAST TR-LUC-GH3-Ant

The dataset TOXCAST was created by in vitro high-throughput screening and included several effects on biochemical endpoints, cellular processes, and phenotypes in humans or animals. The SNN investigation focused on the thyroid function mediated by TR genes regulated by the GH3 cell line. The TR-interacting compounds disrupt thyroid homeostasis. The balanced accuracies of the top-performing SNNs were gathered in Table 5.

#### 2.1.6. Numerical Experiments on BBBP

The blood–brain barrier plays an essential role in protecting the nervous system and maintaining the microenvironment of the brain. Drugs targeting the central nervous system should be able to penetrate the blood–brain barrier. The SNN results on the compounds of the BBBP benchmark in detecting permeability were included in Table 6.

#### 2.1.7. Numerical Experiments on SIDER ISD

The Side Effect Resource dataset describes marketed drugs and their adverse reaction to specific human body systems. Those affecting the immune system were analyzed in the ISD screened by SNNs. Outcomes are summarized in Table 7.

#### 2.1.8. Numerical Experiments on SIDER NSD

Commercial drugs that affect the nervous system as side effects are present in the NSD part of the SIDER dataset. The SNNs evaluated for this type of disorder reached accuracies shown in Table 8.

### 2.2. Hyperparameters Evaluation

The SNN hyperparameters of the best SNN models were reported in Table 9. The models generally had a membrane decay rate between 0.8 and 0.95 and a surrogate gradient of 50 or 75. All the best architectures comprised 1000 neurons, except one with 1200.

To enhance the comparison with previous articles in Section 2.3, the SNN outcomes on the test set have been recomputed in terms of AUC and added to Table 9. The validation set accuracies were included in Appendix A Table A1.

### 2.3. Meta-Analysis

This section is subdivided into two parts: an initial report of previous works running classification on MFs as inputs (Section 2.3.1), and a latter part (Section 2.3.2), including manuscripts adopting different frameworks for in silico toxicity prediction. Section 2.3.3 summarizes in graphical format the comparisons between SNNs and other methodologies from the previous literature.

#### 2.3.1. Previous Literature on Virtual Screenings from MFs

The first comparison was conducted over the results presented by [40] using a random forest (i.e., RF) and Morgan MFs on BBBP (0.909 ± 0.028 AUC), Tox21 (0.819 ± 0.017), SIDER (0.687 ± 0.014), and ClinTox (0.759 ± 0.060). In [41], the authors evaluated MAACS fingerprints over the Tox21 dataset achieving an AUC of 0.805 ± 0.01, an AUC of 0.721 ± 0.004 for BBBP, and an AUC equal to 0.797 ± 0.151 for Clintox, applying an ensemble of decision trees over 5-fold cross-validation. Another paper [42] focused on the Tox21 dataset reporting the outcomes of the in silico toxicity evaluation by five classifiers on Morgan fingerprints: the LightGBM overperformed other classifiers, reaching an AUC of 0.795 on the test set (standard deviation was not reported) for NR-AR. Other classifiers included in the study were random forest (0.777), support vector machines with radial basis function (0.784), extreme gradient boosting (0.777), and a deep neural network with three hidden layers coupled with Adam as optimizer (0.787). This latter work shares with the current investigation the choice of nested CV as a practical approach to keep separated HP tuning and evaluation of the estimators. On NR-ER-LBD, LightGBM reached 0.796, whereas on SR-ATAD5, it fulfilled 0.802 AUC. In [43], the authors analyzed the Tox21 dataset (the 10k version) using multiscale weighted colored graph MFs and compared the results of different classifiers to MAACS MF. The outcomes for the gradient boosting decision tree on MAACS for NR-AR was0.756 AUC, for NR-ER-LBD was 0.788, and for SR-ATAD5 was 0.734. Another work testing several classifiers on Tox21 was [44], reporting on NR-ER-LBD 0.83 AUC employing RF on MAACS MFs, 0.73 AUC with naive Bayes, and 0.78 AUC with probabilistic NN. The authors of [45] generated Morgan MFs for SIDER, BBBP, and Tox21 datasets evaluating the benchmarks by convolutional NN (Table A2). They applied the convolutional NN on single learning tasks for each dataset (similar to what was performed during the current investigation on SNN), or the same NN applied in multitask learning, as in [46], obtaining better results. The authors of [47] evaluated different MFs on Tox21 data with a naïve Bayes classifier, reaching 0.7664 and 0.772 AUC with MAACS MFs for active or inactive compounds on NR-ER-LBD, respectively. The results were raised to 0.8 on the test set combining MFs to a similarity score. The authors used the same scheme to obtain 0.69 on NR-AR and 0.75 on SR-ATAD5. From MAACS MFs and working with an RF classifier, the researchers in [48] reported 0.8151 AUC on SR-ATAD5, adopting a synthetic data generation scheme to solve the class instances’ imbalance. The same group obtained 0.8232 without imbalance adjustments on NR-AR and 0.9133 on NR-ER-LBD with random undersampling.

#### 2.3.2. Previous Literature Applying More Sophisticated Data Representations

In [49], the authors tested SMILES molecular data representations paired to NNs derived from natural language processing on Clintox benchmark. They also verified the outcomes on other frameworks, and statistical learning classifiers with HPs tuned according to the previous literature, as shown in Table A3. In [40], the “directed Message Passing Neural Networks” [50] were attempted on several benchmarks, both as a single estimator or in an ensemble learning configuration (Table A4). The authors of [51] compared pre-trained classification models (self-supervised learning approach) constructed on large molecules databases to MFs generated by autoencoders or traditionally. They evaluated those inputs by statistical learning estimators such as gradient-boosted decision trees, RF, and support vector machines (Table A5). The work of [52] assessed the usage of a transformer-based architecture through interaction scores between each character of the SMILES (aka self-attention); on SIDER, the AUC was 0.858.

#### 2.3.3. Positioning of SNN Results in the Current Body of Knowledge

**Figure 1:**
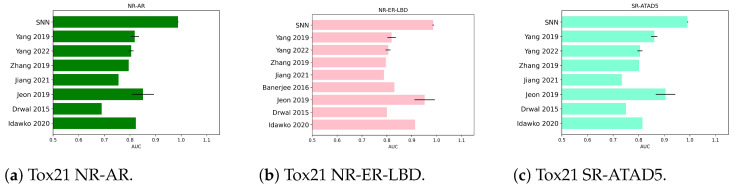
Visual summary of meta-analysis on Tox21. Standard deviation was included if reported in the original papers. AUC values from [40,41,42,43,44,45,47,48].

**Figure 2:**
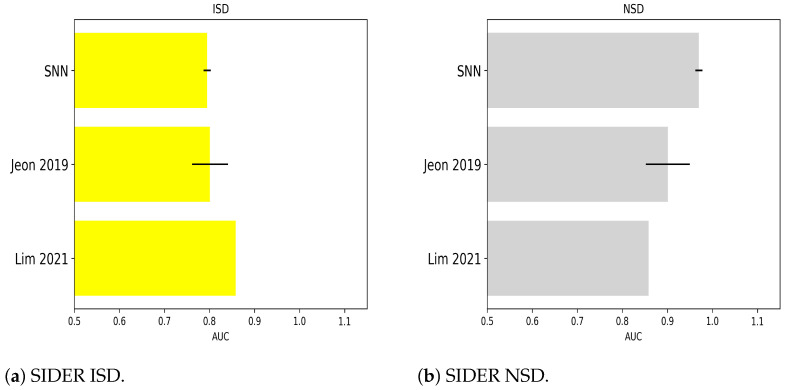
Classification of side effects of chemicals from SIDER dataset in the present and other works. AUC included from [45,52].

**Figure 3:**
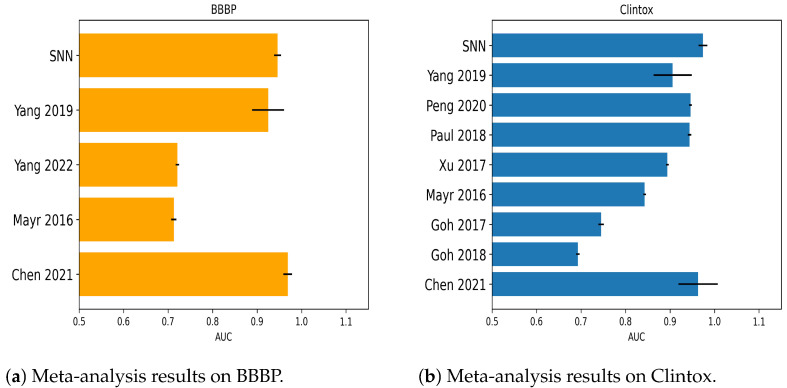
Outcomes for BBBP and Clintox compared to the previous literature. AUC values as reported in [40,41,46,49,51,53,54,55,56].

The following bar plots show AUC obtained by the SNNs’ best models for each dataset employed in the study, referencing previous results as reported in Section 2.3.1 and Section 2.3.2. For Tox21 benchmarks, the comparisons are in Figure 1. For SIDER, Figure 2 collects classification outcomes for the immune and nervous system disorders as side effects of chemicals. Regarding BBBP and Clintox, a meta-analysis of the classifiers’ AUC coming from other sources in the previous literature was included in Figure 3a,b, respectively. Only in the case of Toxcast and probably due to dataset characteristics, more compatible references with the current investigation were needed to prepare a graph (one reference was found).

### 2.4. Scalability of SNNs with Longer MF

During this numerical experiment, MAACS MFs was compared to extended-connectivity fingerprints (i.e., ECFP) calculated with a radius of 2 and 1024 bits in length. Balanced accuracy for BBBP and Clintox datasets was tested using ECFP as input and employing the best models of Table 9. It should be underlined that the number of neurons in the hidden layer or other parameters of the SNN were not re-optimized but left in the same configuration initially calculated for MAACS MF. The BBBP and Clintox datasets were selected due to the high number of cited papers working on these databases, as previously reported in Section 2.3.1 and Section 2.3.2.

Final scores were noted on Table 10: accuracies do not significantly depart from those collected with shorter MF, proving the good scalability of SNNs to longer inputs. Appendix C Table A6 reports the computational times for each dataset.

## 3. Discussion

The numerical experiments demonstrated the application of SNNs for the virtual screening of molecule databases targeting toxicity. Using structural information derived from MF, the SNNs obtained remarkable results compared to the previous literature. The meta-analysis of Section 2.3 showed the consistent performance of SNNs with other high-quality methods previously employed for toxicity prediction. One advantage of SNNs is their ability to handle MFs binary inputs directly without requiring more complex mechanisms to generate learnable input patterns. This investigation is currently the first one employing SNNs for QSAR, proposing this technique as an alternative to classical machine learning or NN methods. Furthermore, exploring neuromorphic computation solutions as alternatives to tackle the von Neumann bottleneck problem [57] could provide new insights to drive future technologies for drug discovery and virtual screening. In von Neumann’s architecture, the chips move information continuously and at high bandwidth between the central processing unit and memory, wasting time and energy. Spiking neuromorphic hardware works differently, being asynchronous and event-based, subject to neuron dynamics and firing timings, with memory located alongside the computational units [58]. Consequently, even if this investigation proposed SNNs employing traditional hardware (laptop computer with Intel i5 CPU and 16Gb RAM), the best computational performance could be achieved by implementing SNNs on neuromorphic silicon-based devices. Regarding computational times, it should be underlined that they were heavily influenced by the number of training epochs required for instructing the network; therefore, the values included in Appendix C Table A6 serve as a reference. Indeed, in light of on-chip learning given by some neuromorphic platforms (Intel Loihi, Darwin neural processing unit, or BrainScaleS), the possibilities of continuously learning new molecules and predicting the target activity in real-time may make the inclusion of computational times irrelevant.

One complication of SNNs mentioned in other sources is the difficulty in setting the right HPs [59]. During the present investigation, the SNNs’ HPs in the best-performing models did not vary significantly (Table 9), reducing the severity of the claim reported in previous papers. In general, the number of HPs for SNN neurons is higher than those in artificial NNs, and more complex neuron models increase the user’s required settings. For example, a slightly more complex neuron than the LIF integrates synaptic conductance that considers the time course of the neurotransmitter released by the pre-synaptic neuron. This addition translates into the introduction of a novel parameter that simulates AMPA (i.e., alpha-amino-3-hydroxy-5-methyl-4-isoxazolepropionic acid), and at a lesser extent, NMDA (i.e., N-methyl-D-aspartate), glutamate receptor activity [60], and modulates the synaptic strength of the LIF. This kind of second-order neurons in MF-based QSAR may be helpful to maintain extensive sparsity coding [61], and they could be evaluated on MFs with a bit length longer than those tested in the current work.

Another remark regarding SNNs that may be of interest for future developments in drug discovery is that the existing architectures do not fully cover all mechanisms of learning, and ongoing research is needed both on the software and hardware side. For example, the authors of [62] recently proposed a method to implement biologically plausible mechanisms to simulate on hardware long–short time dependencies as found in deep learning. This advancement may offer an opportunity for integrated hardware and software solutions to solve complex in silico tasks of medical chemistry. Indeed, one approach followed by some authors [63] was to use recurrent neural networks on bi-dimensional fingerprint-like representations of atoms and bonds. Consequently, technological progress in neuromorphic computing could provide further applications to chemoinformatics, employing systems that reflect the mechanisms of brain activity and thus more explainable than standard “black-box” approaches derived from ANNs.

## 4. Materials and Methods

**Figure 4:**
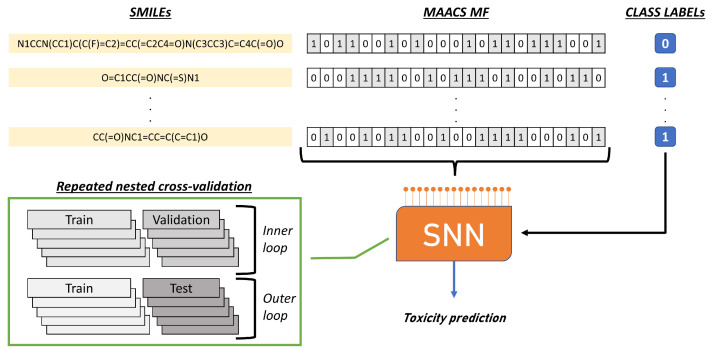
Overview of the procedure during the numerical experiments. The input bit strings were created from SMILES, and a binary label accompanied each instance. The binary sequences were input directly to the SNN, which was evaluated by nested cross-validation with an inner loop for model selection and an outer loop for evaluating the quality of the outcomes.

Five public-domain toxicological datasets were investigated, each evaluated in separate numerical experiments through specific SNNs. All SNNs had in common the neuronal model, the leaky integrate-and-fire (i.e., LIF) [64], and the architecture. An overview of the experimental sequence is illustrated in Figure 4.

### 4.1. Neuron Model

**Figure 5:**
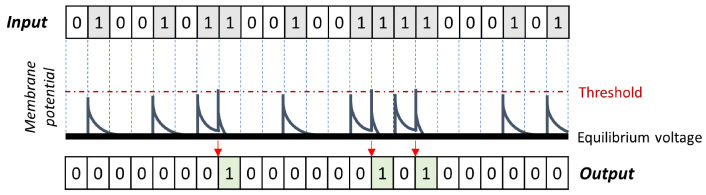
Membrane potential in LIF neurons is modeled receiving a binary train as input and producing a binary output in response: only when membrane potential is over-threshold do LIF neurons fire a spike marked by ones in the output sequence.

Compared to normal cells, biological neurons have peculiar features such as dendrites to capture incoming external signals, an axon to transmit pulses at a distance, and terminal parts called synapses to forward information to the dendrites of other neurons. Through the axon, information is transmitted temporarily varying the concentration gradient of transmembrane ions. This change affects the axon from the beginning to the end, creating a charged ionic flow similar to electric currents in a cable [65]. When a neuron receives inputs from the dendrites, the cell body weighs all ionic charges received, and if the total signal is enough, the cell’s polarity changes. This initial polarization influences the axon membrane gradient that is modified due to the influx of positive ions. Ions rapidly move inside the neuron through ionic channels following gradient concentrations sequentially along the neuron membrane up to the synapse. Once the process is moving toward the synapse, a short hyperpolarization called the refractory period is accompanied by restoring the equilibrium potential by accumulating outside the neuron positively charged ionic species. The complexity of these biological mechanisms is simplified using the single-compartment LIF neurons: the weighted sum of inputs (aka “integration”) triggers ionic changes across the membrane (aka “firing”) [66]. However, the integrator is “leaky” because a small amount becomes lost while integrating inputs over time. The membrane potential fluctuations resulting from the received input sequences are exemplified in Figure 5. The ones in the incoming stimuli are represented as square current pulses that disrupt the ionic equilibrium between axonal cytosol and extracellular fluid, eventually generating a spike (ones in the output sequence). Single perturbations are not enough to activate the neuron response (firing), whereas multiple stimuli overcoming the voltage threshold (red dashed line) elicit a response. After a neuronal spike, the voltage drops rapidly to a hyperpolarization state; later, the neuron is ready to respond again—single stimuli below the threshold return to equilibrium with a slower decay rate than in the case of hyperpolarization. Figure 5 does not explicitly show hyperpolarization but summarizes the different decay to equilibrium in case of firing or not. In the neurophysiological nomenclature, the neuron membrane depolarization responding to an over-threshold input stimulus is called action potential or spike, meaning the neuron is firing and usually marked with a one in a vector describing the neuron activity over time. Mathematically, this could be associated with a Heaviside step function. Periods of below-threshold stimuli are equivalent to zeros, meaning no firing occurs on the neuron membrane. The binary sequences describing neurons’ activity are formally equivalent to bit strings of molecular fingerprints; this aspect allows for a direct usage of MFs as input for SNNs, and no format conversion is needed.

**Figure 6:**
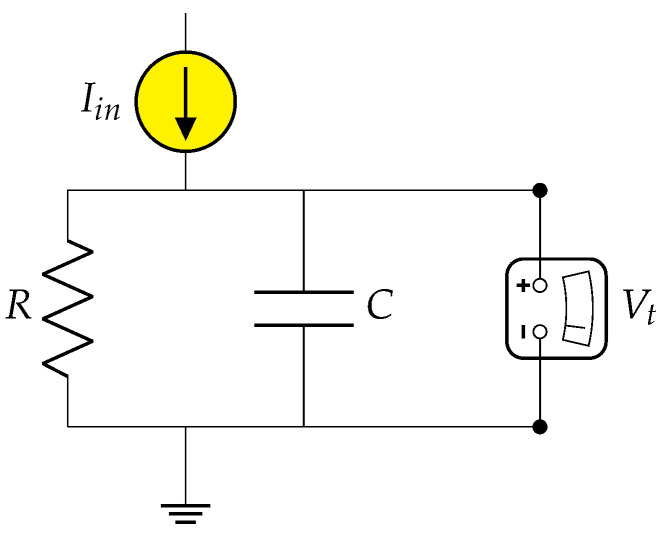
LIF circuit equivalent. A parallel resistor and capacitor represent the neuron, a configuration that could easily describe membrane behavior and, at the same time, is effortlessly implementable on silicon chips.

A first-order low-pass filter could represent a leaky integrator, and from this assumption, the LIF equations are described mathematically in terms of electrical circuits [67]. Ions passing the neuron membrane wall experience a resistance *R* and are subject to a capacitance *C*: the resistance depicts the narrowness of the membrane’s ion channels that slow down the influx, and the capacitance is the lipidic membrane bilayer acting as an insulator (Figure 6).

Considering the time constant of the circuit is τ=RC, Equation (1) describes variations of the LIF membrane potentials as voltage shifts measured by the voltmeter in Figure 6.

(1) $$\tau \frac{dV_{t}}{dt}=-V_{t}+RI_{in}$$

The linear ordinary differential equation could be solved with the forward Euler method for practical application in the SNN. Another characteristic of neurons is the hyperpolarization following a membrane depolarization or firing. This could be achieved with a reset mechanism subtracting a fixed voltage value until reaching equilibrium or simply resetting the voltage to zero; all SNNs were assembled with the first reset option by subtraction.

### 4.2. SNN Architecture

**Figure 7:**
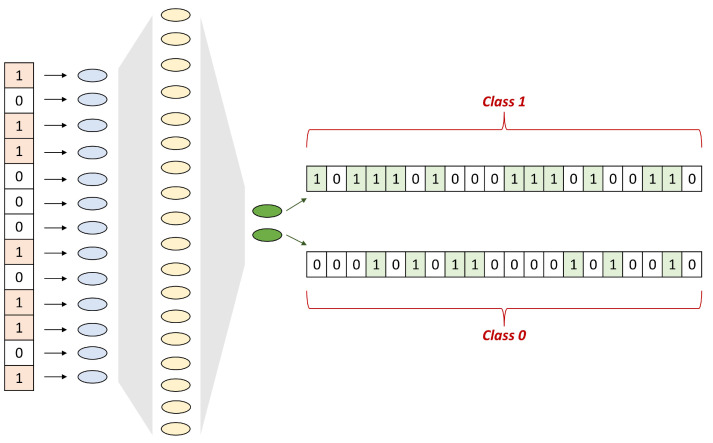
Exemplification of the common SNN architectures. All SNNs employed were shallow with one hidden layer. The fingerprints’ bits were passed to the input layer, whereas the last layer firing counts determined the predicted class.

The SNNs were programmed in Python language, using Torch and derived libraries [68,69]. All networks had the same number of input neurons, equal to the number of bits in the fingerprints’ vectors, one hidden layer, and an output layer of two neurons, as shown in Figure 7. The number of hidden neurons was the subject of investigation during the hyperparameters search.

Class membership was determined by the number of spikes fired by the last two neurons, assigning it to the neuron that fired more spikes. The SNNs were fully connected with input data distributed in mini batches of 64 samples. The subdivision of the input samples in mini-batches avoided the “generalization gap” noticed with large input sample sizes [70]. The discrete nature of the binary vectors employed by SNNs poses the challenge of the lack of differentiability during backpropagation calculations through the chain rule. The solution to this problem has been proposed in [71], by applying a surrogate function that replaces the hard threshold. In other words, during backpropagation, a sigmoid activation is selected with a finite slope instead of computing the derivative of the Heaviside step function (the Dirac delta function).

### 4.3. Model Evaluation and Hyperparameters Tuning

Each SNN had several hyperparameters (i.e., HPs) to be tuned: in the previous literature [72,73,74], this part is usually reported as time-consuming and challenging to perform due to the non-linear relationship between LIF output and HPs. In the current investigation, nested cross-validation (i.e., CV) has been employed to separate the phase of HP optimization and model evaluation. The HP selection was achieved during the inner loop, while the outer loop evaluated model quality. Additionally, the CV has been repeated ten times to analyze model variability and generalization error extensively. Among the HPs searched, the membrane decay rate β, expressed as $V_{t+\Delta t}=\beta \times V_{t}$, and the slope of the surrogate gradient were LIF-specific. More general HPs were the number of hidden neurons, the optimizer type and its parameters, the number of epochs needed for training, and the presence or lack of gradient clipping (i.e., GC). This latter technique is mainly utilized to circumvent exploding gradient issues [75]. Another optimizer HP included was the weight decay (i.e., WD), usually adopted to regularize the network [76].

HP optimization was performed with a random search over a regularly spaced grid of values, except for the learning rate (i.e., LR) with pre-selected intervals. The search range is reported in Table 11. The optimizers implemented in the Python Torch library and surveyed were “Adam” and “Adamax” [77], “Stochastic Gradient (Descent) Optimizer” (i.e., SGO [78]), “Adaptive Gradient Algorithm” (i.e., Adagrad [79]), “Adadelta” [80], “AdamW” [81], and “RMSProp” [82].

### 4.4. Benchmark Datasets Employed in the Study

The compounds explored came from the following repositories, with a summary in Table 12:

- TOXCAST [83], containing results of in vitro toxicological experiments. In particular, the outcomes for “Tox21-TR-LUC-GH3-Antagonist” were considered due to the best sample ratio;
- Tox21 [84], predicting the toxicity on biological targets, including nuclear receptors or stress response pathways. Activities selected were “SR-ATAD5”, “NR-EL-LBD”, and “NR-AR” for the relatively low number of missing entries compared to the others inside the dataset;
- BBBP [85] assessing drug’s blood–brain barrier penetration;
- SIDER [86], employed for predicting drug’s side effects on the immune and nervous systems;
- Clintox [87], containing drugs that failed or passed clinical trials for toxicity.

All of the datasets’ molecules were in the SMILES format with annotated binary labels. The number of instances was unequal in all cases, processed balancing by oversampling the minority class. If class balancing was not performed, the risk was that the classifiers may be over-exposed to the majority class during training. The last column of Table 12 reported the initial instances for the negative and positive classes and the number of examples after equalization inside parenthesis. The results of the numerical experiments were judged through balanced accuracy (i.e., BA). Although equalizing class counts could be sufficient to evaluate models fairly by accuracy, balanced accuracy further ensured unbiased appraisal.

### 4.5. Fingerprints Characteristics

After standardizing and normalizing the functional groups, the SMILES to fingerprint conversion was performed via the RDkit Python library [88]. Preprocessing has been performed with MolVS [89], a software tool included in RDkit. The selected MFsformat was the SMARTS-based implementation of the 166 bits long public MACCS keys.

Another preliminary aspect to verify was the similarity of the molecules inside each dataset. If the compound similarity is high, the classifier may learn representations of analogous molecular structures, affecting the model’s generalization. The heterogeneity of the molecules in each dataset was evaluated through the Jaccard–Tanimoto index and reported as mean values in Table 13. This coefficient demonstrated high reliability in quantifying molecule similarity from MF [90].

In the datasets, the probability of co-occurrences between compounds ranged from 20.8% to 33.5%, or conversely, a mean dissimilarity ranging from 79.2% to 66.5%. The Tox21 family of data and TOXCAST exhibited the most significant heterogeneity between molecules, while BBBP, Clintox, and SIDER collected closer compound types.

### 4.6. Meta-Analysis Criteria

Current classification results on MAACS MFs applying SNNs were compared to the previous literature for each dataset. Journal articles were selected using the Google Scholar engine, integrated with Università del Piemonte Orientale library resources. The dataset name was inserted as keyword together with “molecular fingerprint”. Pre-prints were not considered because they were not yet peer-reviewed. The article list was sorted per publication data, with the most recent on the top, and results scanned in descending order; priority was assigned to works showing outcomes on MAACS MFs for direct comparison with the current investigation. Only articles employing area under the receiver operating characteristic curve (i.e., AUC) or accuracy as evaluation metrics on the test set were considered for compatibility with current outcomes. Another precaution as inclusion criteria involved the selection of studies dividing the samples of the original datasets in random splits (consistent with Molecule Net [85]); other methods, such as scaffold splits, are usually associated with lower AUC due to increased difficulty. In a few cases, the results were presented as figures only, without providing tables to collect numerical information, and consequently, the values could not be retrieved.

## 5. Conclusions

This work witnessed the usage of spiking neural networks for the virtual screening of multiple databases of compounds targeting toxicity. The results were in line with top-notch predictors from the literature, opening the door to the possible usage of neuroscience-inspired artificial intelligence for quantitative structure–activity or property relationship analysis. Future works will further evaluate the applicability of spiking neural networks to other chemoinformatics domains, with the intent of employing an integrated software and hardware neuromorphic architecture.

## Figures and Tables

**Table 1:** Results on Clintox.

Hidden Neur.	β	Grad. Slope	Opt.	LR	WD	GC	Mean BA	Std BA
1000	0.8	75	Adam	0.0001	0	True	97.845	0.661
1000	0.65	50	Adam	1 × 10^−5^	0	True	97.663	0.605
1000	0.6	25	Adam	0.0001	0	True	97.669	0.317
1000	0.95	50	Adam	1 × 10^−5^	0	True	97.530	0.774
1000	0.8	25	Adam	0.0001	0	False	97.339	0.556

**Table 2:** Results on Tox21 NR-AR.

Hidden Neur.	β	Grad. Slope	Opt.	LR	WD	GC	Mean BA	Std BA
1000	0.95	50	Adagrad	0.01	0	False	98.840	0.368
1000	0.95	50	AdamW	0.0001	0	False	98.829	0.232
1000	0.95	50	Adamax	0.002	0	False	98.823	0.369
1500	0.95	50	Adam	5 × 10^−5^	0	False	98.810	0.215
800	0.95	50	Adam	5 × 10^−5^	0	False	98.778	0.241

**Table 3:** Results on Tox21 NR-ER-LBD.

Hidden Neur.	β	Grad. Slope	Opt.	LR	WD	GC	Mean BA	Std BA
1000	0.95	50	Adamax	0.002	0	False	98.491	0.399
1200	0.85	75	Adam	1 × 10^−5^	0	False	98.457	0.270
1000	0.95	50	Adagrad	0.01	0	False	98.456	0.268
1000	0.95	50	Adam	0.0001	0.001	False	98.354	0.373
1000	0.95	50	SGO	0.001	0	False	98.283	0.362

**Table 4:** Results on Tox21 SR-ATAD5.

Hidden Neur.	β	Grad. Slope	Opt.	LR	WD	GC	Mean BA	Std BA
1200	0.85	75	Adam	1 × 10^−5^	0	False	99.055	0.232
1000	0.95	50	Adam	0.0001	0.001	False	98.837	0.257
1000	0.95	50	Adagrad	0.01	0	False	98.818	0.182
1500	0.95	50	SGO	0.005	0.001	False	98.798	0.221
1000	0.95	50	Adam	0.0001	0	False	98.795	0.251

**Table 5:** Results on TOXCAST TR-LUC-GH3-Ant.

Hidden Neur.	β	Grad. Slope	Opt.	LR	WD	GC	Mean BA	Std BA
1000	0.95	50	Adamax	0.002	0	False	91.379	0.819
1000	0.95	50	Adamax	0.002	0.0001	False	91.358	0.417
1200	0.95	50	Adamax	0.002	0	False	91.190	0.877
1000	0.95	50	Adagrad	0.01	0.001	False	91.166	0.391
1200	0.95	50	Adagrad	0.01	0	False	91.138	0.699

**Table 6:** Results on BBBP.

Hidden Neur.	β	Grad. Slope	Opt.	LR	WD	GC	Mean BA	Std BA
1000	0.95	50	Adamax	0.002	0	False	94.481	1.068
1000	0.95	50	SGO	0.005	0	False	94.230	1.342
1000	0.95	50	RMSProp	0.001	0	False	93.883	1.434
1000	0.8	25	Adam	0.0001	0	False	93.838	0.696
1000	0.95	50	Adam	0.0001	0	False	93.651	1.005

**Table 7:** Results on SIDER ISD.

Hidden Neur.	β	Grad. Slope	Opt.	LR	WD	GC	Mean BA	Std BA
1000	0.95	50	Adagrad	0.01	0	False	81.750	2.027
1500	0.95	50	SGO	0.005	0.001	False	81.376	1.998
1000	0.95	50	SGO	0.001	0	False	81.254	3.243
2000	0.95	50	Adagrad	0.01	0	False	80.981	1.905
1000	0.8	25	Adagrad	0.01	0	False	80.937	1.721

**Table 8:** Results on SIDER NSD.

Hidden Neur.	β	Grad. Slope	Opt.	LR	WD	GC	Mean BA	Std BA
1000	0.95	50	SGO	0.001	0.001	False	96.978	0.740
1000	0.95	50	Adadelta	1.0	0	False	96.520	1.197
1000	0.95	50	Adagrad	0.01	0	False	96.515	1.211
1000	0.95	50	AdamW	1 × 10^−5^	0	True	96.450	0.840
1000	0.95	50	Adamax	0.002	0	False	96.374	0.697

**Table 9:** Summary of SNNs’ HPs for top-ranked models.

Dataset	Hidden Neur.	β	Grad. Slope	Mean BA	Std BA	Mean AUC	Std AUC
BBBP	1000	0.95	50	94.481	1.068	0.946	0.008
Clintox	1000	0.8	75	97.845	0.661	0.974	0.01
ISD	1000	0.95	50	81.750	2.027	0.795	0.008
NR-AR	1000	0.95	50	98.840	0.368	0.988	0.002
NR-ER-LBD	1000	0.95	50	98.491	0.399	0.986	0.003
NSD	1000	0.95	50	96.977	0.740	0.97	0.008
SR-ATAD5	1200	0.85	75	99.055	0.232	0.991	0.002
TOXCAST	1000	0.95	50	91.379	0.819	0.912	0.007

**Table 10:** BA of SNNs with MFs of different length as input patterns.

MF Type	BBBP	Clintox
ECFP	93.451 ± 0.684	97.772 ± 0.522
MAACS	94.481 ± 1.068	97.845 ± 0.661

**Table 11:** Parameter grid range during HP tuning. The goal was to identify the HP configuration driving the learning process to reach the best performance on each benchmark dataset.

Hyperparameter	Lower Limit	Upper Limit	Levels
Hidden neurons	500	2000	5
β	0.6	0.95	6
Grad. slope	25	75	3
LR	1 × 10^−5^	0.5	15
WD	0.001	0.05	4

**Table 12:** Datasets information.

Dataset	Activity Studied	Acronym	Instances
Clintox	Toxicity in clinical trials	Clintox	1366 (1366)–112 (1366)
Tox21	Androgen receptor nipple retention	NR-AR	6956 (6956)–309 (6956)
Tox21	Hepatotoxicity	NR-ER-LBD	6605 (6605)–350 (6605)
Tox21	DNA damage	SR-ATAD5	6808 (6808)–264 (6808)
TOXCAST	Thyroid homeostasis disruption	TR-LUC-GH3-Ant ^1^	6170 (6170)–1761 (6170)
BBBP	Blood–brain barrier permeability	BBBP	479 (1560)–1560 (1560)
SIDER	Immune system disorders (iatrogenic toxicity)	ISD	403 (1024)–1024 (1024)
SIDER	Nervous system disorders (iatrogenic toxicity)	NSD	123 (1304)–1304 (1304)

^1^ Murine tissue assays.

**Table 13:** Mean Tanimoto indices of each dataset.

Dataset	Tanimoto Index
Clintox	0.315
NR-AR	0.21
NR-ER-LBD	0.209
SR-ATAD5	0.208
TR-LUC-GH3-Ant	0.211
BBBP	0.335
ISD	0.308
NSD	0.308

## Data Availability

Benchmark datasets used in this paper were public-domain databases of molecules that can be accessed from the MoleculeNet repository [85].

## Abbreviations

The following abbreviations are used in this manuscript:

AUC	Area Under the ROC Curve
BA	Balanced Accuracy
CV	Cross-Validation
ECFPs	Extended-Connectivity Fingerprints
GC	Gradient Clipping
HPs	Hyperparameters
LIF	Leaky Integrate-and-Fire (neuron model)
LR	Learning Rate
MFs	Molecular Fingerprints
ML	Machine Learning
NN	Neural Network (other than SNN)
QSAR	Quantitative Structure–Activity Relationship
QSPR	Quantitative Structure–Property Relationship
RF	Random Forest
ROC	Receiver Operating Characteristic
SGO	Stochastic Gradient Optimizer
SMILES	Simplified Molecular-Input Line-Entry System
SNN	Spiking Neural Network
WD	Weight Decay

## Appendix A

Table A1 reports validation and test set accuracies of the nested CV rounds.

**Table A1:** Balanced accuracy at validation and test sets as percentages.

Dataset	Validation Set	Test Set
Clintox	96.6 ± 0.5	97.8 ± 0.7
NR-AR	98.8 ± 0.4	98.3 ± 0.5
NR-ER-LBD	97.8 ± 0.4	98.5 ± 0.4
SR-ATAD5	95.0 ± 0.6	99.0 ± 0.2
BBBP	92.7 ± 1.3	94.5 ± 1.1
TOXCAST	89.0 ± 1.0	91.4 ± 0.9
NSD	95.8 ± 1.1	97.0 ± 0.8
ISD	78.5 ± 2.3	81.8 ± 2.0

## Appendix B

Table A2 collects the AUC outcomes of [45] as described in Section 2.3.1.

**Table A2:** AUC from single and multiple task learning as shown in [45].

Benchmark	Single	Multiple
ISD	0.670 ± 0.038	0.801 ± 0.040
NSD	0.820 ± 0.083	0.901 ± 0.049
NR-AR	0.810 ± 0.027	0.851 ± 0.043
NR-ER-LBD	0.898 ± 0.040	0.952 ± 0.041
SR-ATAD5	0.844 ± 0.033	0.905 ± 0.038
BBBP	0.713 ± 0.006	

Table A3 shows results as reported in [49] (Section 2.3.2).

**Table A3:** AUC on ClinTox from [49].

Method	Ref.	Clintox
TOP	[49]	0.946 ± 0.003
CheMixNet	[53]	0.944 ± 0.004
DeepAOT	[54]	0.894 ± 0.003
DeepTox	[46]	0.843 ± 0.003
Chemception	[55]	0.745 ± 0.006
SMILES2Vec	[56]	0.693 ± 0.004
RF		0.769 ± 0.002
SVM		0.751 ± 0.002
KNN ^1^		0.698 ± 0.003

^1^ k—nearest neighbors algorithm.

Table A4 presents AUC values from [40] (Section 2.3.2).

**Table A4:** AUC from the numerical experiments in [40].

Benchmark	D-MPNN ^1^	D-MPNN (Ensemble)
BBBP	0.913 ± 0.026	0.925 ± 0.036
Tox21	0.845 ± 0.015	0.861 ± 0.012
SIDER	0.646 ± 0.016	0.664 ± 0.021
Clintox	0.894 ± 0.027	0.906 ± 0.043

^1^ directed Message Passing NN.

Table A5 displays scores as detailed in [51] (Section 2.3.2).

**Table A5:** Best models AUC in [51] for Clintox and BBBP applying different MF sets.

MF Type	Features	BBBP	Clintox
SSLP-FPs	C [51]	0.949 ± 0.016	0.963 ± 0.044
SSLP-FPs	CP [51]	0.953 ± 0.009	0.939 ± 0.047
SSLP-FPs	CPZ [51]	0.946 ± 0.015	0.941 ± 0.035
Auto-FPs		0.969 ± 0.01	0.95 ± 0.037
ECFC2	1024 bits	0.92 ± 0.015	0.821 ± 0.058
ECFC2	2048 bits	0.919 ± 0.021	0.833 ± 0.053
ECFC2	512 bits	0.913 ± 0.019	0.831 ± 0.056
ECFC4	1024 bits	0.914 ± 0.024	0.782 ± 0.052
ECFC4	2048 bits	0.916 ± 0.021	0.784 ± 0.053
ECFC4	512 bits	0.908 ± 0.025	0.801 ± 0.049
ECFC6	1024 bits	0.907 ± 0.029	0.77 ± 0.054
ECFC6	2048 bits	0.911 ± 0.026	0.75 ± 0.059
ECFC6	512 bits	0.9 ± 0.032	0.77 ± 0.048

## Appendix C

Table A6 reports the average number of seconds taken during the ten repetitions of nested CV ECFP analysis on BBBP and Clintox datasets. Computations were accomplished on commodity hardware (laptop computer with Intel i5 CPU and 16Gb RAM). Furthermore, the table includes the number of training epochs as a parameter directly affecting the computational time.

**Table A6:** Computational times for ECFP.

Dataset	Computational Time (s)	Training Epochs
Clintox	4109.55	300
BBBP	1522.74	100

In general, computational times could be heavily influenced by several factors (for example, the number of training epochs, the batch size, the number of parallel jobs on the computer, the use of CPU or GPU, etc.); thus, they should be evaluated considering all these aspects. Additionally, SNN architectures should be programmed on neuromorphic boards to improve computational performance because adapting SNN on traditional hardware may only highlight some advantages of this technique.
